# Supplementary material for: A compact X-ray source via fast microparticle streams
Source: Commun Eng. 2024 Nov 15;3:171. doi: 10.1038/s44172-024-00323-z (PMC11568210; doi:10.1038/s44172-024-00323-z)
Supplement: Supplementary file 2 — Supplementary information [file 44172_2024_323_MOESM2_ESM.pdf]

Supplementary Information

Supplementary Discussion: Müller–Oosterkamp surface heating model

The performance of state-of-the-art rotating anode tubes is limited by focal track erosion. The Müller–Oosterkamp theory is typically employed in order to specify the input power of rotating anode tubes for different focal spot sizes[37]. It leads to a simple maximum temperature rise expression that reads $\Delta T_{\mathrm{M}} = 2(q_0/k)\,\sqrt{}[(\alpha_{\mathrm{d}} \cdot W)/(\pi \nu)]$, where k is the thermal conductivity, α_d the thermal diffusivity, ν the local anode velocity and q_0 the incident heat flux. If the focal spot width W is proportional to its length, as typical for medical imaging, this ultimately translates to the proportionality of the permitted input power $P \propto W^{3/2}$. There are several approximations behind the Müller–Oosterkamp theory which are necessary to convert the exact numerical heat transfer problem to an analytically solvable two-dimensional boundary value problem. The most critical approximation is the surface heating assumption, which can be violated for narrow focal spots and high tube voltages[38], e. g. for diagnostic magnification radiography with focal spots smaller than 0.2 mm[38]. In fact, at high electron energies, the characteristic depth of electron penetration is no longer much smaller than the focal spot dimensions, which implies that volumetric heating should be considered. Thus, the Fourier heat flux surface boundary condition now becomes trivial, and a volumetric heat source term should be added to the heat conduction equation. Whitaker acknowledged the necessity for volumetric heating and introduced the reasonable assumptions that are needed to set-up an analytically solvable two-dimensional boundary value problem[30]. The most critical approximation is the oversimplifying assumption that the volumetric heating can be approximated by an exponential decay with respect to the depth. This leads to an analytical solution with the aid of Laplace transforms and to a closed-form expression for the maximum temperature rise ΔT_{W}. Ultimately, Whitaker derived a tube voltage dependent power correction factor $\Lambda = \Delta T_{\mathrm{W}}/\Delta T_{\mathrm{M}}$, based on the additional assumption that, during the residence of the electron beam, a maximum temperature rise in the focal spot would be permitted that is independent of the tube voltage. However, this assumption is not adequate from a thermal fatigue perspective. The mechanisms of tungsten cracking and track erosion will change with the location and extension of the heated zone within the target[39,40]. Hence, they are expected to depend on the tube voltage. The vendors of diagnostic X-ray tubes do not publish voltage dependent power data that are typically validated in life cycle tests.

37. Müller, A. A “spinning target X-ray generator” and its input limit. *Proc. Roy. Soc. A* 125, 507 (1929). https://doi.org/10.1098/rspa.1929.0181
38. Dietz, K. A Rotating-Anode X-Ray Tube with Microfocus. Röntgenstrahler, Siemens AG, Erlangen, Germany 6, 1–10 (1982). Remark: It is unclear if the focal spot width of 0.2 mm discussed in the paper denotes real or standardized nominal dimensions according to the medical standard IEC 60336[16]. The nominal dimension for a real 0.2 mm wide focal spot using standardized limits of 15 % intensity of the line-spread function would be be 0.15.
39. Hirai, T., Pintsuk, G., Linke, J., Batilliot, M. Cracking failure study of ITER-reference tungsten grade under single pulse thermal shock loads at elevated temperatures. *J. Nucl. Mater.* 390391, 751 (2009). https://doi.org/10.1016/j.jnucmat.2009.01.313
40. Rieth, M., Dudarev, S. L., Gonzalez de Vicente, S. M., Aktaa, J., Ahlgren, T., Antusch, S., Armstrong, D. E. J., Balden, M., Baluc, N., Barthe, M.-F., Basuki, W. W., Battabyal, M., Becquart, C. S., Blagoeva, D., Boldyryeva, H., Brinkmann, J., Celino, M., Ciupinski, L., Correia, J. B., De Backer, A., Domain, C., Gaganidze, E., Garcia-Rosales, C., Gibson, J., Gilbert, M. R., Giusepponi, S., Gludovatz, B., Greuner, H., Heinola, K., Höschen, T., Hoffmann, A., Holstein, N., Koch, F., Krauss, W., Li, H., Lindig, S., Linke, J., Linsmeier, Ch., López-Ruiz, P., Maier, H., Matejicek, J.,

Mishra, T. P., Muhammed, M., Muñoz, A., Muzyk, M., Nordlund, K., Nguyen-Manh, D., Opschoor, J., Ordás, N., Palacios, T., Pintsuk, G., Pippan, R., Reiser, J., Riesch, J., Roberts, S. G., Romaner, L., Rosiñski, M., Sanchez, M., Schulmeyer, W., Traxler, H., Ureña, A., van der Laan, J. G., Veleva, L., Wahlberg, S., Walter, M., Weber, T., Weitkamp, T., Wurster, S., Yar, M. A., You, J. H., Zivelonghi, A. Recent progress in research on tungsten materials for nuclear fusion applications in Europe. *J. Nucl. Mater.* 432, 482 (2013). https://doi.org/10.1016/j.jnucmat.2012.08.018

Supplementary Notes: Maximal target input power for tungsten

The mass heat capacity $c_W(T)$ of tungsten rises substantially with temperature, notably close to the melting point $T_{m,W}$[33]. Unlike compact targets that would unacceptably erode, microparticle targets may be operated in this regime. When the temperature T of microparticles rises from T_0 upstream of the interaction region to $T_{m,W}$ at the exit after their residence time Δt, the maximal permitted areal target input energy density p_{max} is

$$p_{max} = \frac{\rho_W}{\Delta t \cdot \zeta_W(E_e, d_{peak})} \left[\int_{T_0}^{T_{m,W}} c_W(T)\ dT + c_{fusion} \right].$$

The temperature dependence of the mass density ρ_W shall be ignored in this context, as it is below 5% in the range from ambient to the melting point. $\int_{T_0}^{T_{m,W}} c_W(T)\ dT$ is numerically evaluated based on Fig. 5b of ref.[33]. $\zeta_w(E_e, d_{peak})$ is evaluated by Monte Carlo simulation[32], Extended Data Fig. 2a.

Compared with increasing the temperature from 100 °C to the melting point, phase change by melting would add up to 48 % mass heat of fusion c_{fusion} to the heat capacity. Melting might be permitted for non-aggregating dilute targets if a long tube geometry would allow microparticles to re-solidify by in-flight heat radiation cooling before hitting a stopping device.

A reduced macroscopic atom density in a microparticle stream causes a reduced macroscopic mass density $\rho_{\mu P}$ such that $\rho_{\mu P}/\rho_W$ cancels out from the above expression for p_{max}. Maximal stopping power is found at the depth $d_{peak} \cdot \rho_W / \rho_{\mu P}$. With the length of the electron beam cross section L_{cs}, its width W_{cs}, and the microparticle velocity $v_{\mu P}$, Δt amounts to $W_{CS} / v_{\mu P}$. The input power is $P_{input} = U_{tube} \cdot I_{input} = W_{CS} \cdot L_{CS} \cdot p_{max}$, whereby I_{input} is the primary input electron current and U_{tube} the tube voltage, is then

$$P_{input} = \frac{\rho_W \cdot L_{cs} \cdot v_{\mu p}}{\zeta_W(E_e, d_{peak})} \left[\int_{T_0}^{T_{m,W}} c_W(T)\ dT + c_{fusion} \right].$$

Supplementary Methods 1: Required Target Density and Derating Factors

An electron beam impinging on a standard compact target defines line spread functions (LSF) of X-ray intensity along axes normal to the central X-ray beam. As usual, this beam may be skewed with respect to the target surface by the target angle, α. The line spread function in the length direction LSF_L is determined in an evaluation plane that comprises the central X-ray beam and the central line of the electron beam. The LSF_L is measured with a line camera and represents the incident X-ray intensity measured along all lines parallel to the central X-ray beam, and integrated normal to the plane. The line spread function in the width direction LSF_W is evaluated in a similar way, but oriented orthogonal to the length direction. According to the medical imaging standard[16], the dimensions of the focal spot are the distances between the abscissa values where LSF_L and LSF_W undercut 15% of their respective peak values. In the typical case of compact targets and isotropic electron current densities, where electron scattering can be ignored, this simply projects the electron beam current density in the cross section with the target to a plane normal to the central beam. While the intensity distribution along the width is directly mapped, the cross-sectional focal spot length L_{CS} seemingly shrinks to the X-ray optical focal spot length $L = L_{CS} \cdot \tan(\alpha)$.

In contrast to compact targets, the mass density reduction in microparticle targets may yield large electron scattering ranges and radiation “coronae" reaching to a substantial distance from the impact cross section of the primary electron beam. Apertures, that might be positioned proximal to the interaction region to eliminate radiation from coronae, would be subject to excessive electron impact and are not practical. Furthermore, the X-ray conversion efficiency would suffer. The only practical course of action for the realization of the desired focal spot dimensions consists in reducing the cross section of the primary electron beam to accommodate margins for X-ray coronae within the desired focal spot. Length and width must be treated separately. At small target angles, the X-ray corona along the depth axis nearly directly increases the focal spot length. Due to the projection, lateral electron scattering in the length direction has a smaller impact on the LSF_L, while a corona in the width direction straightforwardly widens the LSF_W. Supplementary Methods Fig. 1a and b allow an assessment of the upper limits for filtration dependent X-ray coronae that will, in practical cases, be of the order of 60 – 75% of the stopping power coronae, as shown in Extended Data Fig. 2b.

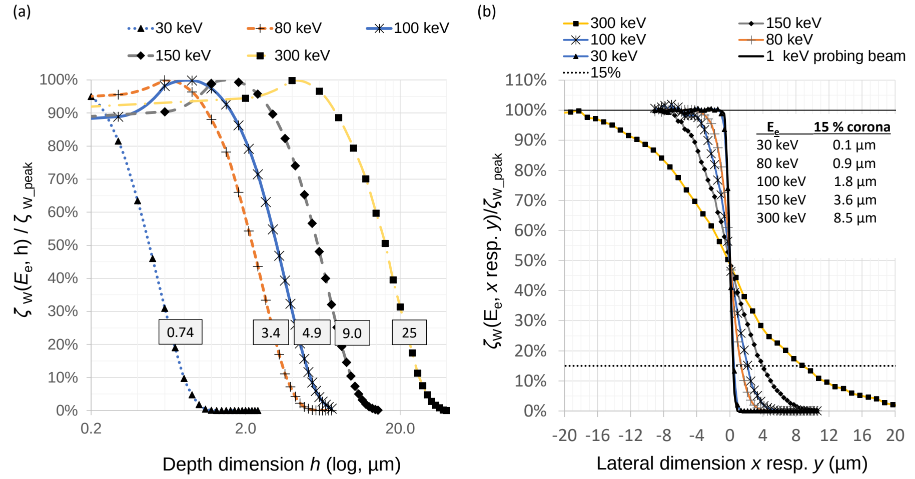

Supplementary Methods Figure 1 Scattering margins and X-ray coronas. (a) The results of CASINO Monte Carlo simulations for the stopping power functions $\zeta_W(E_e,h)$ of compact tungsten at selected primary electron energies E_e in the range of 30 – 300

keV. The stopping power functions are all normalized over their respective incident energy dependent maxima. The depth axis h is logarithmic. The depth corona limits d_{15}, i.e., the loci where the stopping power functions attain 15% of their maximum, are designated in the boxes; 10^5 primary events for 30 and 300 keV, 10^6 for 80 and 150 keV, $5x10^6$ for 100 keV. (b) The results of CASINO simulations for the lateral electron scattering at an edge (at $x = 0$) of a semi-infinite electron beam at selected primary electron energies in the range of 30 – 300 keV. The lateral X-ray corona margins l_{15}, i.e., the loci where the functions attain 15% of the maximum, are listed inside the table to the right. A 1 keV probing beam was utilized in the simulation to characterize the nearly rectangular electron beam current density.

Formatted: Font: Not Italic

Formatted: Font: Not Italic

Supplementary Methods Fig. 1(a) assesses the upper limits of coronae d_{15} in the depth direction, as derived from the stopping power function, while Supplementary Notes Fig. 1 (b) quantifies the lateral scattering. The region at the edge of a simulated electron beam with a sharp edge is depleted from electrons, whereas the external region is populated. For the quantitative analysis, the simulated stopping power per voxel has been summed over the depth direction and then projected to the target surface. Under the reasonable assumption that the stopping power can represent the X-ray intensity also for this lateral case, at all incident electron energies, the lateral X-ray corona is assessed by measuring from the edge of the electron beam (set to $x = 0$) up to the point where the projected stopping power undercuts the 15% line of the respective value in the centre of a large focal spot (100% intensity). CASINO requires a circular electron beam cross section. To minimize the errors, only a small rectangular target area with a non-vanishing tangential extension reaching into this beam was evaluated. A probing beam of 1 keV electron energy with an assumed infinitely small scattering spread ("stopping power corona") was simulated with the same geometry aiming to delineate the simulation specific uncertainties and the location of the zero point, that was taken at the crossing of the 50% line for each curve. The reduction of the electron beams cross section, that may be necessary for the accommodation of the combined X-ray coronae, will require the reduction of the permitted power rating of the X-ray source. Supplementary Table lists the reduction factors that should be applied to the gain factor $G_{0.3}$ for a sample focal spot of nominal 0.3 and 8° target angle (standard IEC 60336[16]) depicted in Fig. 4 of the main article.

It is worth emphasizing that the upper limits of the X-ray coronae in the lateral and depth directions are adopted from the stopping power function ζ. This leads to an overestimation of the corona size of the X-ray intensity of the filtered beam that defines the standardized LSFs. Strong filtration, e.g., by the object, will further shorten the line-spread functions. Thus, Supplementary Table 1, ignoring any filtration, lists the worst case. It can be discerned that a microparticle target density of 10% of the compact tungsten density would still suffice to deliver high output. Therefore, it is advised to simultaneously fulfil both, often contradicting, requirements: sufficient microparticle powder supply and high velocity. A target density of 1% and smaller may result in excessive coronae for 150 kV and 300 kV tube voltages. Thus, such cases are represented by zero percent in the table.

Supplementary Methods 2: The SPEKPY V2 toolkit and X-ray Intensity Depth Curves

SpekPy V2 is a validated software toolkit that models X-ray spectra and carries out spectrum-related postprocessing. It includes the most advanced physics models available in similar purpose software[34]. The top-level spectral models are elaborated in ref.[41] and have been validated in refs.[34,42], while the underlying physics models are evaluated in refs.[43,44]. In brief, the depth resolved electron frequency distributions in solid target materials (differential in energy and direction), that have been pre-computed with the PENELOPE code[45], serve as the input data to SpekPy. Then, the bremsstrahlung fluence at a given emission angle is calculated, based on tabulations of the bremsstrahlung cross-section (differential in emission energy) and on tabulations of the so-called "shape-function" of the angular distribution of bremsstrahlung emission. The characteristic X-ray fluence (L- & K-lines) is generated based on frequency depth distributions that are again pre-computed with PENELOPE, including direct (via electron impact ionization) and indirect (via bremsstrahlung) sources of atomic relaxation. Both bremsstrahlung and characteristic emissions are corrected for the fluence "self-filtration" by the target itself, as well as an optional amount of added filtration.

An example of X-ray spectrum prediction is shown in Supplementary Methods Fig. 2, together with a comparison to measurements made at a National Standards facility[46]. As illustrated in the figure, the SpekPy toolkit can provide a fluence spectrum at a given emission direction that is integrated over all depths of emission in the target. For the purposes of the current work, the Python codebase was modified to allow the extraction of the pre-integrated fluence spectra contributions. These could then be converted to intensity spectra and integrated over X-ray energy to provide intensity-depth curves for X-ray emissions from solid targets (instead of fluence-energy curves). The toolkit options selected for the study concern a tungsten target, an 8° target angle, the X-ray linear attenuation coefficients of PENELOPE (with the default re-normalised photoelectric effect contribution) and the "SIM" shape-function for bremsstrahlung emission, i.e., the leading term of the standard 2BN angular distribution[41]. A filtration of 2.5mm of Al was applied in addition to the intrinsic self-filtration of the fluence spectra by tungsten.

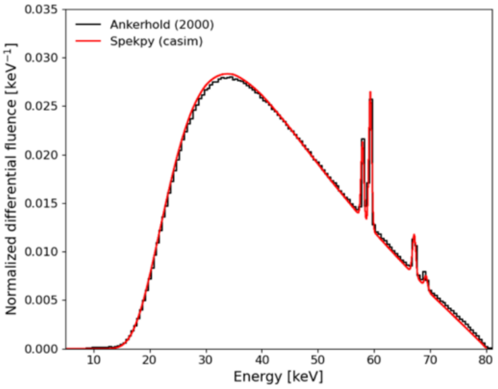

Supplementary Methods Figure 2 Comparison of the SpekPy V2 predictions (casim option)[34] with the measurements of Ankerhold[46] for an RQR6 radiographic spectrum. The areas beneath the curves are normalized to unity. The bin width for the SpekPy calculations was 0.1 keV with a Gaussian filter (σ = 0.25 keV) applied to represent the detector energy resolution.

In the present study, it has been assumed that the X-ray intensity-depth curve from an ensemble of microparticles corresponds to the X-ray intensity-depth curve from a solid target (as generated from the SpekPy toolkit) that has been re-scaled by the ratio of the average granular

medium mass density to the mass density of the compact target. Fig. 5 (b) of the main manuscript depicts the resulting intensity-depth curve for an incident electron kinetic energy of 100 keV and a compact tungsten target with a 100% packing fraction.

41. Omar, A., Andreo, P., Poludniowski, G. A model for the energy and angular distribution of x rays emitted from an x-ray tube. Part I. Bremsstrahlung production. *Med. Phys.* 47, 4763 (2020). DOI: https://doi.org/10.1002/mp.14359
42. Omar, A., Andreo, P., Poludniowski, G. A model for the energy and angular distribution of x rays emitted from an x-ray tube. Part II. Validation of x-ray spectra from 20 to 300 kV. *Med. Phys.* 47, 4005 (2020). https://doi.org/10.1002/mp.14360
43. Omar, A., Andreo, P., Poludniowski, G. Performance of different theories for the angular distribution of bremsstrahlung produced by keV electrons incident upon a target. *Radiat. Phys. Chem.* 148, 73 (2018). DOI: https://doi.org/10.1016/j.radphyschem.2018.02.009
44. Omar, A., Andreo, P., Poludniowski, G. A model for the emission of K and L X-rays from an X-ray tube. *Nucl. Instrum. Meth. Phys. Res. B* 437, 36 (2018). DOI: https://doi.org/10.1016/j.nimb.2018.10.026
45. F. Salvat, PENELOPE 2018: Code system for Monte Carlo simulation of electron and photon transport, (OECD Publishing, Paris, 2019)
46. U. Ankerhold, PTB Report Dos-34 (Braunschweig, Germany: Physikalisch-Technische Bundesanstalt; 2000)

Supplementary Methods 3: Model settings for the Casino Monte Carlo simulations

All Monte Carlo electron transport simulations reported in this study were performed with the CASINO 3.3.0.4 software[32,47]. This code is tailor made for the investigation of electron backscattering, electron transmission, secondary electron emission and electron dissipation in condensed matter at the energy ranges of interest, making it the preferred software for our purposes. In CASINO, the total and differential elastic scattering cross-sections are adopted from the ELSEPA code[48], which numerically solves the Dirac equation in a spherically symmetric interaction potential that considers the screening of the nuclear field by the surrounding electrons. In case of a bare Coulomb interaction, this procedure would yield the Mott scattering cross-section. The ELSEPA interaction potential includes an electrostatic term with contributions from the nucleus and the surrounding electron density, an exchange term within a local approximation, a correlation-polarization term described by a Buckingham potential and an imaginary absorption term that describes loss from elastic to inelastic channels. In CASINO simulations, a database generated from ELSEPA for input electron energies up to 500 keV is interpolated. It is also important to note that pre-calculated ELSEPA tables are also utilized in the PENELOPE code and the GEANT4 code. In addition, the mean ionization - excitation losses are described by a semi-empirical stopping power formula that incorporates energy variability into the mean ionization potential[48]. For high electron energies, this formula naturally collapses to the standard Bethe expression. For low electron energies, this formula avoids the well-known positive stopping power pathology of the Bethe expression and is constructed so that the results of multi-shell Bethe versions are accurately reproduced. Finally, the generation of internal secondary electrons is treated with a hybrid model for inelastic scattering, where fast secondary generation is based on the Møller full QED differential cross-section and slow secondary generation is based on linear response theory. The following model settings were employed in all simulations reported in this study: (i) Use of room temperature mass density. (ii) Secondary electron fine tuning details: Maximum order generated: 10, residual energy loss: 0.4 eV. Secondary electron production was always selected to improve accuracy. However, emitted secondaries were not included in the backscattering and transmission yields. (iii) Minimum energy of simulated electrons equal to the work function. (iv) Use of the lagged Fibonacci pseudo-random number generator. (v) New direction cosine calculation as in the NIST MONSEL code.

Supplementary Methods 4: Validation of the Casino Monte Carlo simulation software

We benchmarked CASINO 3.3.0.4 predictions against relevant reliable experimental results. We used 10^6 primary test events (primary electrons) for each MC simulation and achieved a statistical variance smaller than 0.1%, assuming Poisson statistics. Given the spherical transparent microparticles of interest, the validation exercise focused on the normal and oblique electron backscattering yields of semi-infinite planar targets as well as the normal electron backscattering yields and electron transmission yields of transparent films. Since tungsten (Z = 74) data were only available for normal electron incidence on bulk planar targets, tantalum (Z = 73) and gold (Z = 79) were utilized as proxy materials. This is justified by the fact that the backscattering and transmission yields mainly depend on the atomic number in the keV electron energy range of interest. The CASINO predictions for the normal incidence electron backscattering yield of bulk semi-infinite tungsten plates have been compared with the experimental results of Hunger and Küchler[50] in the 4-40 keV energy range (eight data points, accessed from tabulations), the measurements of Reimer and Tollkamp[51] in the 3-30 keV energy range (six data points, accessed from tabulations in Joy's[49] electron-solid interaction database) as well as the experimental results of Heinrich[52] in the 10-49 keV energy range (five data points, accessed from tabulations in Joy's[49] electron-solid interaction database). The deviation between experiments and simulations is satisfactory in the relevant energy range, see Supplementary Methods Fig. 3.

Formatted: Superscript

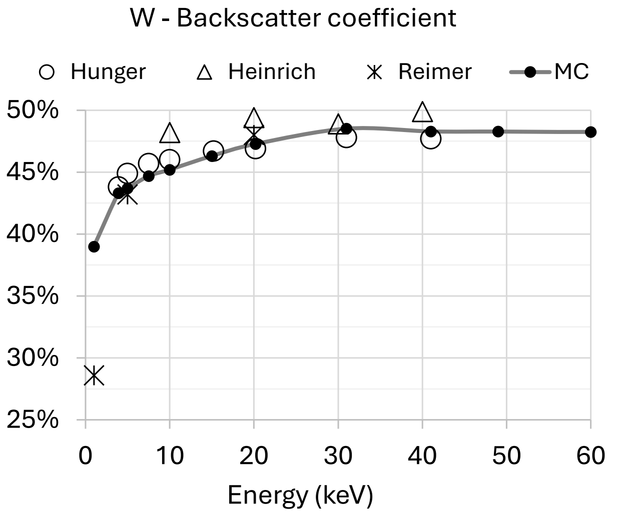

Supplementary Methods Figure 3 Normal incidence electron backscattering yield of bulk tungsten slab experiments. Refs. 49,51,50 (from left) vs. CASINO 3.3.0.4 (MC, dots, lines added to guide the eye)

The CASINO predictions for the normal and oblique incidence electron backscattering yield of bulk tantalum and gold plates were benchmarked against the measurements of Neubert and Rogaschewski[53] at 20; 40; 60 keV in the 0-80°incidence range (ten data points per incident angle, accessed from tabulations). In the case of gold, the normal incidence experiments of Drescher et al.[54] in the 10-100 keV energy range (seven data points, accessed from tabulations) have also been included. Considering the experimental uncertainties at near grazing angles as well as the fact that these measurements were not carried out in ultra-high vacuum with provisions for in-situ surface cleaning[55], the agreement is judged to be satisfactory. Examples are shown in Supplementary Methods Fig. 4 and 5.

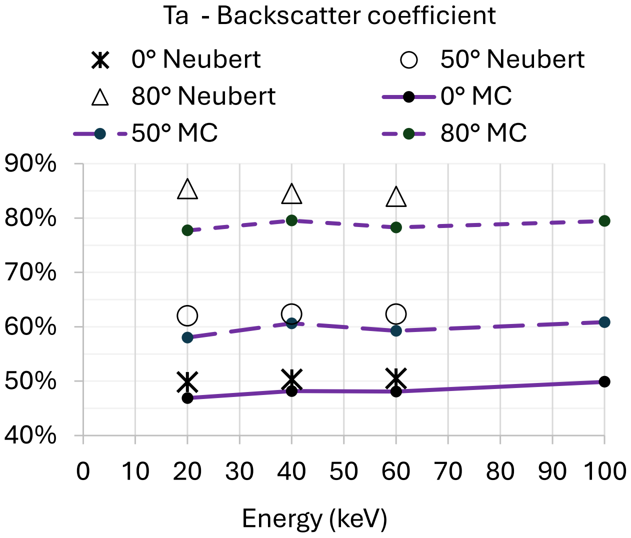

Supplementary Methods Figure 4 Normal and oblique incidence electron backscattering yield of bulk tantalum slabs; CASINO (dots, lines added to guide the eye) vs. experiments[53] (lines added to guide the eye)

The CASINO predictions for the normal incidence electron backscattering yield and electron transmission yield of transparent gold films have been benchmarked against the measurements of Reimer and Drescher[56] for 10-500 nm foil thickness in the 10-100 keV energy range. The data were extracted with the aid of software and were offset-corrected by linearly extrapolating to the limit of zero foil thickness, where the backscattering yield is 0% and the transmission yield is 100%. The CASINO simulations featured a 1 nm wide incident electron beam normal on a cylindric gold slab of varying thickness with a model radius of 100 µm that was chosen much larger than that the CSDA (continuous slowing down approximation) range for 300 keV electrons in gold, to minimize errors from electron diffusion at the slab edges.

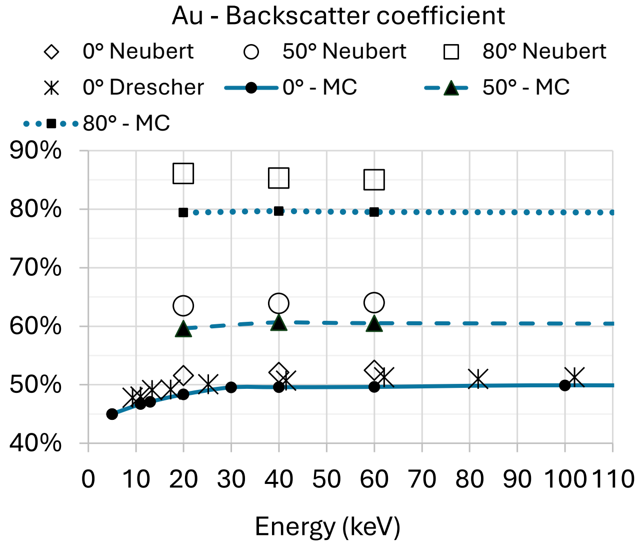

Supplementary Methods Figure 5 Normal & oblique incidence electron backscattering yield of bulk gold slabs; CASINO (MC, dots, lines added to guide the eye) vs. experiments, refs.[53,54]

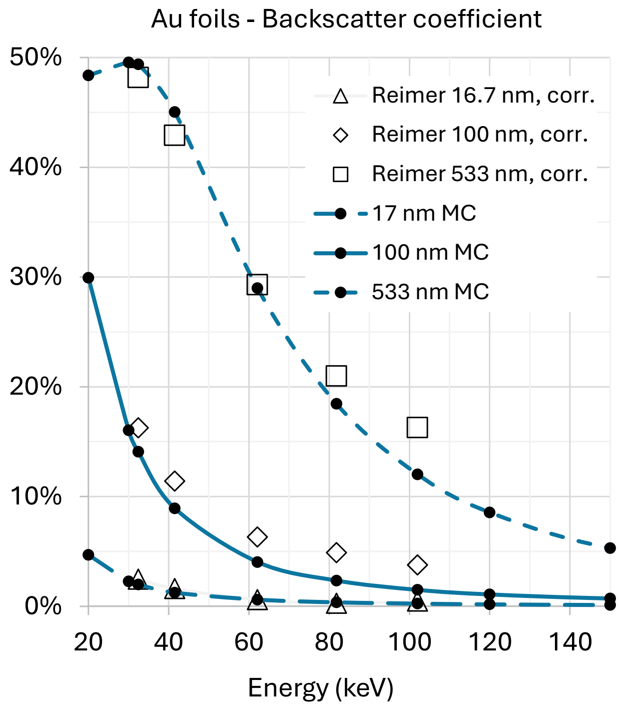

Supplementary Methods Figure 6 Normal incidence electron backscattering yield of transparent gold foils; CASINO (dots, lines added to guide the eye) vs. experiments[56]

The CASINO simulations can satisfactorily reproduce both the backscattering and the transmission yields regardless of the film thickness and the incident electron energy, see Supplementary Methods Fig. 6 and 7. An Excel file with the source data for Supplementary Methods Fig. 3–7 is provided. Therein, the procedure for the offset correction of the experimental data for the transparent film backscattering and transmission yields is described in further detail.

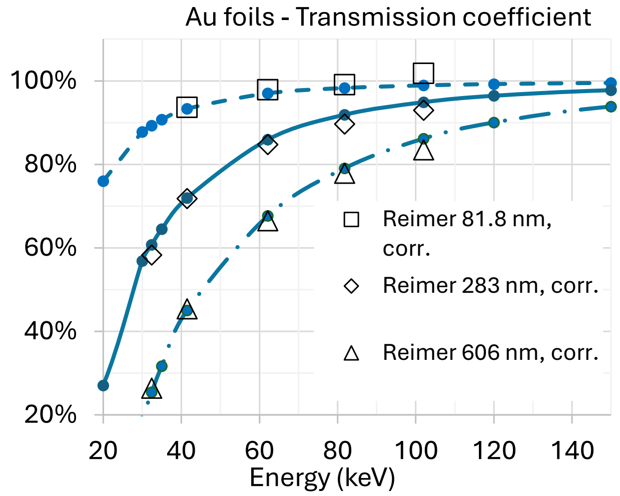

Supplementary Methods Figure 7 Normal incidence electron transmission yield of transparent gold foils; CASINO (MC, dots, lines added to guide the eye) vs. experiments[56]

47. Hovington, P., D. Drouin, D., Gauvin, R., CASINO: A new Monte Carlo code in C language for electron beam interaction—Part I: Description of the program. Scanning 19, 1 (1997). DOI: https://doi.org/10.1002/sca.4950190101

Field Code Changed

48. Salvat, F., Jablonski, A., C. J. Powell, C.J. Elsepa—Dirac partial-wave calculation of elastic scattering of electrons and positrons by atoms, positive ions and molecules (New Version Announcement. *Comput. Phys.Commun.* 165, 157 (2005). DOI: https://doi.org/10.1016/j.cpc.2020.107704
49. Joy, D. C. & Luo, S. An empirical stopping power relationship for low-energy electrons. *Scanning* 11, 176–180 (1989). https://doi.org/10.1002/sca.4950110404
50. Hunger, H.-J. & Küchler, L. Measurements of the electron backscattering coefficient for quantitative EPMA in the energy range of 4 to 40 keV. *Physica Status Solidi (a)* 56, K45–K48 (1979). https://doi.org/10.1002/pssa.2210560157

Field Code Changed

51. Reimer, L. & Tollkamp, C. Measuring the backscattering coefficient and secondary electron yield inside a scanning electron microscope. *Scanning* 3, 35–39 (1980). https://doi.org/10.1002/sca.4950030105
52. Heinrich, Kurt. F. J. Electron probe microanalysis by specimen current measurement, cited after D. C. Joy: A database of electron-solid interactions, revision #08-1, April 2008, *Scanning* 17, p. 270-275 (1995). in *4th Cong . Int. sur l'Optique des Rayons X et Microanalyse (eds. Castaing, R., Deschamps, P. & Philibert, J.)* 159 (Herman, 1966).
53. Neubert, G. & Rogaschewski, S. Backscattering coefficient measurements of 15 to 60 keV electrons for solids at various angles of incidence. *Phys. Stat. Sol. (a)* 59, 35–41 (1980). DOI: https://doi.org/10.1002/pssa.2210590104
54. Drescher, H., Reimer, L., Seidel, H. Rückstreukoeffizient und Sekundärelektronen-Ausbeute von 10–100 keV-Elektronen und Beziehungen zur Raster-Elektronenmikroskopie. *Z. Angew. Physik* 29, 331 (1970)
55. Tolias, P. On secondary electron emission and its semi-empirical description. *Plasma Phys. Control. Fusion* 56, 123002 (2014). DOI: https://doi.org/10.1088/0741-3335/56/12/123002

56. Reimer, L. & Drescher, H. Secondary electron emission of 10-100 keV electrons from transparent filmy of Al and Au. *J. Phys. D: Appl. Phys* 10, 805–815 (1977). DOI: https://doi.org/10.1088/0022-3727/10/5/022

Supplementary Table

Supplementary Notes Table 1 Worst-case reduction factors to be applied to the power ratings for the given tungsten mass densities. The reduction factors accommodate for X-ray coronae from electron scattering. They naturally depend on the tube voltage and the microparticle stream density. A sample focal spot 0.3 with 8° target angle is assumed (nominal according to the standard IEC 60336[16]).

Tube voltage (kV)	100% (compact)	74% (close packing)	10%	1%	0.1%
30	1	1	0.99	0.88	0
80	1	1	0.95	0.45	0
100	1	1	0.93	0.19	0
150	1	1	0.87	0	0
300	1	1	0.62	0	0
